# A phase 4 study of nilotinib in Korean patients with Philadelphia chromosome‐positive chronic myeloid leukemia in chronic phase: ENESTKorea

**DOI:** 10.1002/cam4.1450

**Published:** 2018-03-25

**Authors:** Junghoon Shin, Youngil Koh, Seo Hyun Yoon, Joo‐Youn Cho, Dae‐Young Kim, Kyoo‐Hyung Lee, Hyeong‐Joon Kim, Jae‐Sook Ahn, Yeo‐Kyeoung Kim, Jinny Park, Sang‐Kyun Sohn, Joon Ho Moon, Yoo Jin Lee, Seonghae Yoon, Jeong‐Ok Lee, June‐Won Cheong, Kyoung Ha Kim, Sung‐Hyun Kim, Hoon‐Gu Kim, Hawk Kim, Seung‐Hyun Nam, Young Rok Do, Sang‐Gon Park, Seong Kyu Park, Sung Hwa Bae, Hun Ho Song, Dong‐Yeop Shin, Doyeun Oh, Min Kyoung Kim, Chul Won Jung, Seonyang Park, Inho Kim

**Affiliations:** ^1^ Department of Internal Medicine Seoul National University Hospital Cancer Research Institute Seoul National University College of Medicine Seoul Korea; ^2^ Department of Clinical Pharmacology and Therapeutics Seoul National University Hospital Seoul Korea; ^3^ Department of Internal Medicine Asan Medical Center Seoul Korea; ^4^ Department of Internal Medicine Chonnam National University Hwasun Hospital Hwasun Korea; ^5^ Department of Internal Medicine Gachon University Gil Medical Center Incheon Korea; ^6^ Department of Internal Medicine Kyungpook National University Hospital Daegu Korea; ^7^ Clinical Trials Center Seoul National University Bundang Hospital Seongnam Korea; ^8^ Department of Internal Medicine Seoul National University Bundang Hospital Seongnam Korea; ^9^ Department of Internal Medicine Severance Hospital Seoul Korea; ^10^ Department of Internal Medicine Soonchunhyang University Seoul Hospital Seoul Korea; ^11^ Department of Internal Medicine Dong‐A University Hospital Busan Korea; ^12^ Department of Internal Medicine Gyeongsang National University Hospital Jinju Korea; ^13^ Department of Internal Medicine Ulsan University Hospital Ulsan Korea; ^14^ Department of Internal Medicine VHS Medical Center Seoul Korea; ^15^ Department of Internal Medicine Keimyung University Dongsan Medical Center Daegu Korea; ^16^ Department of Internal Medicine Chosun University Hospital Gwangju Korea; ^17^ Department of Internal Medicine Soonchunhyang University Bucheon Hospital Bucheon Korea; ^18^ Department of Internal Medicine Daegu Catholic University Medical Center Daegu Korea; ^19^ Department of Internal Medicine Kangdong Sacred Heart Hospital Seoul Korea; ^20^ Department of Internal Medicine Korea Cancer Center Hospital Seoul Korea; ^21^ Department of Internal Medicine CHA Bundang Medical Center Seongnam Korea; ^22^ Department of Internal Medicine Yeungnam University Medical Center Daegu Korea; ^23^ Department of Internal Medicine Samsung Medical Center Seoul Korea

**Keywords:** CML, molecular response, nilotinib, prognosis

## Abstract

Although nilotinib has improved efficacy compared to imatinib, suboptimal response and intolerable adverse events (AEs) limit its effectiveness in many patients with chronic myeloid leukemia in chronic phase (CML‐CP). We investigated the 2‐year efficacy and safety of nilotinib and their relationships with plasma nilotinib concentrations (PNCs). In this open‐label, multi‐institutional phase 4 study, 110 Philadelphia chromosome‐positive CML‐CP patients were treated with nilotinib at a starting dose of 300 mg twice daily. Molecular responses (MRs) and AEs were monitored for up to 24 months. The 24‐month cumulative MR ^4.5^ rate was evaluated as the primary endpoint. Plasma samples were collected from 94 patients to determine PNCs, and the per‐patient mean was used to categorize them into four mean PNC (MPNC) groups. Cumulative MR rates and safety were compared between groups. With a median follow‐up of 22.2 months, the 24‐month cumulative MR ^4.5^ rate was 56.2% (95% confidence interval, 44.0%–8.3%), and the median time to MR ^4.5^ was 23.3 months. There were no significant differences in the cumulative rates of major molecular response, MR ^4^, and MR ^4.5^ between MPNC groups. One patient died due to acute viral hepatitis, and two developed hematological or cytogenetic relapse, while no progression to accelerated or blast phase was observed. Safety results were consistent with previous studies with no new safety signal identified. Across the MPNC groups, there was no significant linear trend in the frequency of AEs. Nilotinib is highly effective for the treatment of CML‐CP with manageable AEs. The measurement of PNC has no predictive value for patient outcomes and is thus not found to be clinically useful. This study is registered with clinicaltrials.gov, Number NCT03332511.

## Introduction

Chronic myeloid leukemia (CML) is a clonal hematopoietic stem cell disorder caused by a reciprocal balanced translocation between the *ABL1* locus and the *BCR* regions in the long arms of chromosome 9 and 22, respectively, which results in the formation of the *BCR‐ABL1* fusion gene. The unregulated kinase activity of the *BCR‐ABL1* oncoprotein mediates autophosphorylation and activation of multiple downstream signaling pathways and results in the uncontrolled proliferation and reduced apoptosis of CML cells 1. With the introduction of imatinib, the first‐generation tyrosine kinase inhibitor (TKI), the prognosis was revolutionized, with the 10‐year survival reaching ~85% and 10‐year relative survival over 90% 2, 3.

Nilotinib is a second‐generation TKI with improved efficacy in terms of an earlier and deeper molecular response, lower rates of progression to accelerated or blast phase, fewer CML‐related deaths, and fewer treatment‐emergent *BCR‐ABL1* mutations, when compared to imatinib 4, 5, 6. Based on the promising results of the landmark phase 3 trial, along with risks of adverse events (AEs) comparable with imatinib, its use was approved for the treatment of newly diagnosed CML in chronic phase (CML‐CP) and imatinib‐resistant or imatinib‐intolerant CML in chronic or accelerated phase 4. However, even with this highly effective agent, there are still many patients for whom the therapeutic response is inadequate, or toxicity is limiting the treatment 7. Therefore, there is room for further optimization of the current CML therapy.

Increasing the dose of nilotinib is associated with a dose‐proportional increase in steady‐state serum levels, and the major AEs of nilotinib are known to occur in a dose‐dependent manner 8. Despite the administration of a uniform dosage, considerable interpatient variability in the serum concentration of nilotinib has been observed, implying that drug exposure may differ substantially between patients even when taking identical doses 9. Serum concentration of nilotinib was shown to affect time to response and progression, making it a surrogate marker for prognosis and the severity of certain AEs 9, 10. Based on these observations, we hypothesized that the optimal plasma level of nilotinib that is sufficient to achieve adequate clinical response while not generating major AEs could be elucidated by the analysis of combined clinical and pharmacokinetic data. Here, we report the results of Evaluating Nilotinib Efficacy and Safety in Clinical Trials‐Korea (ENESTKorea) which evaluated the 2‐year efficacy and safety of nilotinib treatment, and the relationship between the plasma nilotinib concentration (PNC) and clinical outcomes using prospectively collected patient data and plasma samples of CML‐CP patients treated with nilotinib in South Korea.

## Patients and Methods

### Patient eligibility

ENESTKorea was a phase 4, multi‐institutional, single‐arm, open‐label study investigating the efficacy and safety of nilotinib at the currently approved dose (300 mg twice daily) in adult patients diagnosed as Philadelphia chromosome (Ph)‐positive CML‐CP. The diagnosis was confirmed by cytogenetic analysis of at least 20 bone marrow metaphase cells, performed locally using standard methods, within the 6 months before enrollment. See supporting information for a list of exclusion criteria (Data S1).

### Treatment and assessment

Eligible patients were enrolled within 14 days of screening and followed for up to 24 months. Patients were treated with nilotinib at a starting dose of 300 mg twice daily, with a 12‐h interval. Transient interruption of treatment was recommended at the occurrence of grade 3 or 4 hematological AEs (except for anemia) or of grade 2–4 nonhematological AEs. Upon improvement, treatment was resumed at the original dose, resumed at a decreased dose, or discontinued indefinitely, depending on the severity and frequency of occurrence of AEs (Data S2). Investigators were encouraged to attempt to escalate the dose to the starting level if patients were free from dose‐limiting AEs after a four‐week period of reduced dose treatment. If, however, they showed no recovery, despite the interruption of treatment, or if treatment failed, the administration was permanently discontinued. Treatment failure was defined as follows: no complete hematological response (CHR) at 3 months, Ph > 65% at 6 months, no partial cytogenetic response (PCyR: Ph ≤ 35%) at 12 months, no complete cytogenetic response (CCyR: no Ph observed by cytogenetic analysis) at 18 months, loss of CHR, PCyR, or CCyR, or progression to the accelerated or blast phase.

The *BCR*‐*ABL1* transcript type was determined by multiplex polymerase chain reaction at baseline 11. Quantitative real‐time polymerase chain reactions (qRT–PCRs) were performed at the central laboratory (BML, Daejeon, South Korea) every 3 months, for the quantification of *BCR‐ABL1* fusion transcripts, standardized to the international scale (*BCR*‐*ABL1* ^IS^) 12. AEs were prospectively assessed and recorded throughout the study treatment, at maximum of three‐month intervals. They were graded according to the Common Terminology Criteria for Adverse Events, version 4.0 13.

### PNC measurement

Plasma samples were collected every 3 months, for up to 12 months, to determine nilotinib concentrations. This time frame was longer than the sufficient time to reach a steady state (achieved by day 8) 14. Because the mandatory collection of plasma samples was specified in an amendment to the study protocol in March 2014, fewer measurements were performed for some patients. To obtain a trough PNC level, patients were encouraged to visit the study center before 10:00 am on the day of plasma sampling and not to take nilotinib before sampling on that day.

Plasma concentrations of nilotinib were determined using liquid chromatography–tandem mass spectrometry (Agilent 1260 HPLC system and Agilent 6460 Triple Quadrupole; Agilent Technologies, Inc., Santa Clara, CA). The analyte was separated with a xBrigeTM C18 column (3.5 *μ*m particle size, 2.1 × 50 mm; Waters, Milford, MA). The mobile phase used a mixture of 10 mmol/L ammonium acetate, with 0.1% formic acid in distilled water and 0.1% formic acid in acetonitrile, under gradient conditions. The calibration curve was linear over the range of 5–5000 ng/mL (*r* ^2^ ≥ 0.9998). The precision results of quality control samples were all <3.481% and the mean accuracy within ±4.82% of nominal values.

### Study endpoints

The primary endpoint was the cumulative rate of molecular response 4.5 (MR^4.5^; *BCR*‐*ABL1* ^IS^ ≤0.0032%) by 24 months. Secondary endpoints included the cumulative rates of major molecular response (MMR; *BCR‐ABL1* ^IS^ ≤0.1%) and molecular response 4 (MR^4^; *BCR‐ABL1* ^IS^ ≤0.01%) by 12 and 24 months; rates of MMR, MR^4^, and MR^4.5^ at 3, 12, and 18 months; time to MMR, MR^4^, and MR^4.5^; progression‐free survival (PFS); overall survival (OS); and safety. Disease progression was defined as the development of an accelerated or blast phase or the loss of complete hematological or cytogenetic response. PFS was defined as the time from enrollment to documented disease progression or death from any cause. OS was defined as the time from enrollment to death from any cause. Data regarding outcomes were collected only during the study treatment; after discontinuation, data collection was also terminated.

### Statistical analysis

Landmark analyses of primary and secondary endpoints included the intention‐to‐treat (ITT) population (all enrolled patients). For calculation of the response rates “at” designated time points, patients were considered responders only if response assessment at a specified time point indicated achievement of the response. The 95% confidence intervals were calculated using the Clopper–Pearson method. Cumulative response rates and time to responses were presented as time‐to‐response graphs, using a cumulative incidence function 15. For calculation of the cumulative response rates, patients who achieved a response at, or before, a specified time point were treated as responders “by” that time point. Dropouts due to treatment failure, AEs, death from any cause, or withdrawal of consent were considered as competing risks of the response. If these patients dropped out before achieving a response, they were counted as nonresponders thereafter. Patients who were lost to follow‐up due to transfer to another institution, or without a documented reason, were censored on the last qRT–PCR assessment date. PFS and OS were estimated using the Kaplan–Meier method. If no event was recorded, patients were censored on the last follow‐up date.

Correlations between PNCs and clinical outcomes were analyzed based on the patients with available PNC data, who were categorized into quartile groups according to their per‐patient arithmetic mean PNC (MPNC). Categorical and continuous variables were compared between MPNC groups, using Fisher's exact tests and one‐way analyses of variance, respectively. Cumulative rates of MMR, MR^4^, and MR^4.5^ were compared using Gray's test 16. A Fine and Gray subdistribution hazards model was constructed to estimate the hazard ratios for molecular responses in each MPNC group, after adjusting for demographic and clinical variables 17. Only complete cases with no missing data were used for modeling. The Cochran–Armitage trend test was used to test whether the frequency of AEs had a linear trend across the MPNC groups.

Tests were two‐tailed, a *P*‐value <0.05 was considered statistically significant, and no adjustment was made for multiple comparisons. R version 3.4.1 (R Foundation for Statistical Computing, Vienna, Austria) was used for computation.

## Results

### Patient characteristics and PNC

**Figure 1:**
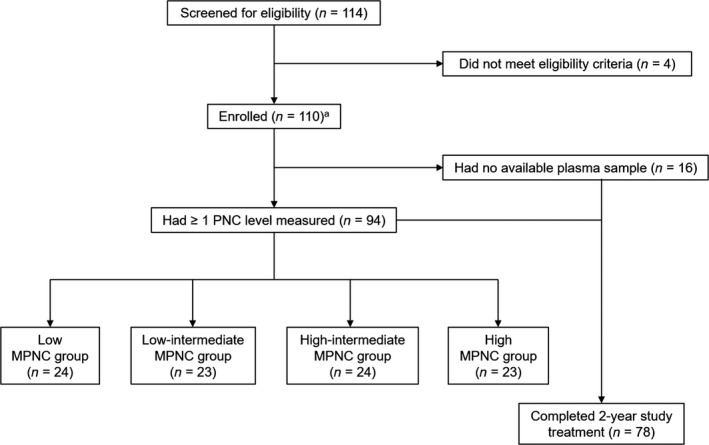
CONSORT diagram. ^a^These patients comprise the intention‐to‐treat population. PNC, plasma nilotinib concentration; MPNC, mean plasma nilotinib concentration.

Between May 2013 and November 2014, 110 CML patients from 20 institutions in South Korea were enrolled in ENESTKorea (Fig. 1). The median age was 55 years at enrollment (Table 1). Prior treatment with hydroxyurea and imatinib accounted for 58 (52.7%) and 1 (0.9%) patient(s), respectively. In total, 78 (71%) patients completed the full 2 years of study treatment (median follow‐up duration, 22.2 [range, 0–26.2] months). Dropout reasons included AEs (*n* = 9), treatment failure (*n* = 3), withdrawal of consent (*n* = 7), and transfer to another institution (*n* = 6). No reason was documented in seven patients.

**Table 1:** Patient characteristics

Variables	ITT population (*N* = 110)^a^	Low MPNC (*N* = 24)	Low‐intermediate MPNC (*N* = 23)	High‐intermediate MPNC (*N* = 24)	High MPNC (*N* = 23)	*P*‐value
Median age at enrollment (range), years	55 (18–83)	57.5 (23–78)	58 (19–80)	54.5 (28–83)	51 (27–68)	0.418
Age group, *n* (%)						
<65	83 (75.5)	17 (70.8)	18 (78.3)	16 (66.7)	21 (91.3)	0.45
≥65	27 (24.5)	7 (29.2)	5 (21.7)	8 (33.3)	2 (8.7)	
Sex, *n* (%)						
Male	71 (64.5)	19 (79.2)	16 (69.6)	11 (45.8)	17 (73.9)	0.083
Female	39 (35.5)	5 (20.8)	7 (30.4)	13 (54.2)	6 (26.1)	
Anthropometry, median (range)						
Height, cm	165 (146–188)	167.5 (149–178)	168 (150–188)	166 (146–182)	161 (149–185)	0.251
Body weight, kg	64 (43–100)	62.5 (49–79)	63 (46–100)	65 (46–89)	65 (46–90.4)	0.849
BMI, kg/m^2^	23.8 (18–30.3)	22.8 (18.6–26.6)	23.7 (18–28.3)	25 (19.1–27.8)	24.1 (19.7–30.3)	0.188
ECOG performance status, *n* (%)						
0	78 (70.9)	16 (66.7)	17 (73.9)	20 (83.3)	15 (65.2)	0.558
1	28 (25.5)	5 (20.8)	6 (26.1)	4 (16.7)	8 (34.8)	
2	1 (0.9)	1 (4.2)	0 (0)	0 (0)	0 (0)	
Not recorded	3 (2.7)	2 (8.3)	0 (0)	0 (0)	0 (0)	
Prior treatment, *n* (%)						
Hydroxyurea	58 (52.7)	13 (54.2)	15 (65.2)	12 (50)	11 (47.8)	0.655
Imatinib	1 (0.9)	0 (0)	0 (0)	0 (0)	0 (0)	
Neither	50 (45.5)	10 (41.7)	8 (34.8)	12 (50)	12 (52.2)	
Not recorded	1 (0.9)	1 (4.2)	0 (0)	0 (0)	0 (0)	
Completion of study treatment, *n* (%)						
Completed	78 (70.9)	17 (70.8)	18 (78.3)	21 (87.5)	22 (95.7)	0.11
Discontinued	32 (29.1)	7 (29.2)	5 (21.7)	3 (12.5)	1 (4.3)	

a The sum of numbers of four MPNC groups (94) is not equal to the number of the number of the ITT population (110) as 16 patients had no PNC data.

ITT, intention to treat; MPNC, mean plasma nilotinib concentration; BMI, body mass index; ECOG, Eastern Cooperative Oncology Group; MPNC, mean plasma nilotinib concentration.

Plasma samples for PNC measurements were available for 94 patients (all four measurements for 49 patients, 3 for 21, 2 for 13, and 1 for 11 patients). PNC ranged from 44.2 to 5053 ng/mL with the median value of 1270.5 ng/mL (Fig. S1). Inter‐ and intra‐individual variations in the PNC are shown in Figure S2, showing approximately double the amount of variation explained by the interindividual rather than by the intra‐individual differences (65.2% and 34.8% of the total variance, respectively). There was a slight increasing trend in PNC levels with time after enrollment (Fig. S3). After averaging the PNC levels for each patient, the MPNC levels ranged from 437.4 to 3311.7 ng/mL with the median at 1309.5 ng/mL. The quartiles of MPNC levels were used for classifying the 94 subjects into four groups: the low (437–1055 ng/mL), low‐intermediate (1055–1311 ng/mL), high‐intermediate (1311–1667 ng/mL), and high (1667–3312 ng/mL) MPNC groups (Fig. 1). Overall, demographical and clinical characteristics were balanced across MPNC groups (Table 1).

### Molecular response and survival

**Figure 2:**
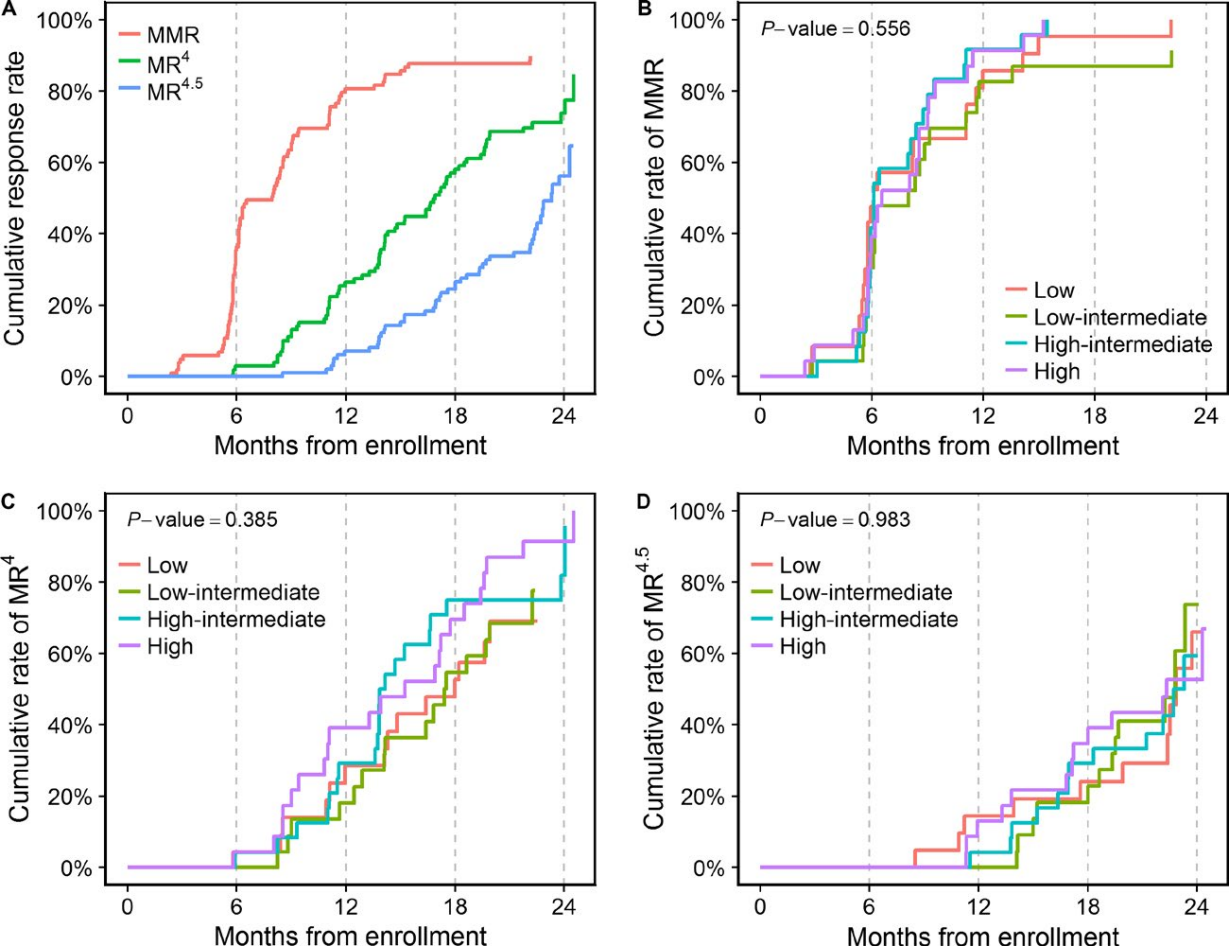
Cumulative molecular response rates in the intention‐to‐treat population (A) and cumulative MMR (B), MR ^4^ (C), and MR ^4.5^ (D) rates in each of the four MPNC groups. MMR, major molecular response; MR ^4^, molecular response 4; MR ^4.5^, molecular response 4.5; MPNC, mean plasma nilotinib concentration.

Among patients in the ITT population, the cumulative rate of MR^4.5^ by 24 months was 56.2% (95% confidence interval [CI], 44%–68.3%), and the median time to MR^4.5^ was 23.3 months (Fig. 2A). Cumulative rates of MMR and MR^4^ by 12 months were 80.6% (95% CI, 72.8–88.4%) and 26.4% (95% CI, 17.6%–35.2%), respectively. By 24 months, the cumulative rate of MMR could not be estimated as the longest follow‐up duration among nonresponders (with respect to MMR) was 22.1 months, while the cumulative rate of MR^4^ was 73.8% (95% CI, 64–83.7%). Rates of MMR, MR^4^, and MR^4.5^ at 3, 12, and 18 months are summarized in Table 2. The median time to MMR and MR^4^ was 8 and 16.9 months, respectively. All patients who completed the 2‐year study treatment achieved MMR (*n* = 78), while 87.2% (*n* = 68) and 59% (*n* = 46) of them achieved MR^4^ and MR^4.5^.

**Table 2:** Rates of MMR, MR^4^, and MR^4.5^ at 3, 12, and 18 months (intention‐to‐treat population; *n* = 110)

	3 months	12 months	18 months
MMR, *n* (%)	6 (5.5)	74 (67.3)	78 (70.9)
95% CI	2–11.5	57.7–75.9	61.5–79.2
MR^4^, *n* (%)	0 (0)	25 (22.7)	56 (50.9)
95% CI	0–3.3	15.3–31.7	41.2–60.6
MR^4.5^, *n* (%)	0 (0)	8 (7.3)	24 (21.8)
95% CI	0–3.3	3.2–13.8	14.5–30.7

MMR, major molecular response; MR^4^, molecular response 4; MR^4.5^, molecular response 4.5; CI, confidence interval.

The median time to MMR, MR^4^, and MR^4.5^ was 6.1, 18, and 22.9 months, respectively, in the low MPNC group; 8, 17.4, and 22.8 months in the low‐intermediate MPNC group; 6.1, 13.9, and 22.7 months in the high‐intermediate MPNC group; and 6.5, 15.2, and 22.3 months in the high MPNC group. In the nonparametrical comparison, there were no significant differences in the cumulative rates of MMR, MR^4^, and MR^4.5^ among the four MPNC groups (Fig. 1B–D). After excluding two cases with incomplete data, the multivariable Fine and Gray subdistribution hazards models also revealed no significant differences in the cumulative rates of MMR, MR^4^, and MR^4.5^ between groups (Table 3).

**Table 3:** Fine and Gray subdistribution hazards model for the cumulative rates of MMR, MR^4^, and MR^4.5^

MPNC groups	MMR^a^		MR^4^ a		MR^4.5^ b	
	HR (95% CI)	*P*‐value	HR (95% CI)	*P*‐value	HR (95% CI)	*P*‐value
Low	1		1		1	
Low‐intermediate	0.75 (0.37–1.51)	0.41	1.11 (0.55–2.23)	0.77	1.18 (0.53–2.62)	0.68
High‐intermediate	1.23 (0.65–2.32)	0.52	1.25 (0.61–2.54)	0.54	0.9 (0.4–2.02)	0.8
High	1.18 (0.63–2.18)	0.61	1.75 (0.91–3.37)	0.094	1.23 (0.53–2.86)	0.63

a Models for the MMR and MR^4^ were adjusted for the age at enrollment (<65 vs. ≥65), sex, ECOG performance status, and prior treatment with hydroxyurea.

b The model for the MR^4.5^ was adjusted for the same variables except for the ECOG performance status since including it resulted in overfitting.

MMR, major molecular response; MR^4^, molecular response 4; MR^4.5^, molecular response 4.5; MPNC, mean plasma nilotinib concentration; HR, hazard ratio; CI, confidence interval; ECOG, Eastern Cooperative Oncology Group.

Throughout the study period, hematological or cytogenetic relapse occurred in only 2 (1.8%) patients and death in only 1 (0.9%). No progression to accelerated or blast phase was observed. The median PFS and OS were not reached by study completion. The 24‐month PFS rate and OS rate were 96.6% (95% CI, 92.8–100%) and 99% (95% CI, 97–100%), respectively. No statistical comparison of survival was performed among the four MPNC groups due to the small number of events.

### Safety

Among the ITT population, AEs (of any grade) were reported in 95 (86.4%) patients and grade 3–5 AEs in 36 patients (32.7%; Table 4). Most nonhematological AEs were of grade 1 or 2. The most common nonlaboratory AEs were skin rashes and QT interval prolongation, reported in 38 (34.5%) and 36 (32.7%) patients, respectively. Grade 3 and 4 nonlaboratory AEs included abdominal pain (*n* = 3), QT interval prolongation (*n* = 2), cerebral infarction (*n* = 2), infectious colitis (*n* = 2), skin rash (*n* = 1), acute viral hepatitis (*n* = 1), acute pancreatitis (*n* = 1), unstable angina (*n* = 1), mechanical ileus (*n* = 1), meningitis (*n* = 1), and congestive heart failure (*n* = 1). One patient died due to acute hepatitis A virus infection.

**Table 4:** Adverse events reported in the ITT population (*N* = 110)

Adverse events	Any grade	Grade 1–2	Grade 3–5
Total, *n* (%)	95 (86.4)	94 (85.5)	35 (31.8)^a^
Nonlaboratory abnormalities, *n* (%)^b^			
Skin rash	38 (34.5)	37 (33.6)	1 (0.9)
QT interval prolongation	36 (32.7)	34 (30.9)	2 (1.8)
Headache	21 (19.1)	21 (19.1)	0 (0)
Fatigue	19 (17.3)	19 (17.3)	0 (0)
Abdominal pain	16 (14.5)	13 (11.8)	3 (2.7)
Pruritus	15 (13.6)	15 (13.6)	0 (0)
Myalgia	14 (12.7)	14 (12.7)	0 (0)
Anorexia	12 (10.9)	12 (10.9)	0 (0)
Nausea	9 (8.2)	9 (8.2)	0 (0)
Alopecia	6 (5.5)	6 (5.5)	0 (0)
Hematological abnormalities, *n* (%)			
Anemia	65 (59.1)	58 (52.7)	7 (6.4)
Thrombocytopenia	38 (34.5)	28 (25.5)	10 (9.1)
Leukopenia	27 (24.5)	23 (20.9)	4 (3.6)
Neutropenia	22 (20)	13 (11.8)	9 (8.2)
Febrile neutropenia	2 (1.8)	NA	2 (1.8)
Biochemical abnormalities, *n* (%)^b^			
Hypocalcemia	62 (56.4)	61 (55.5)	1 (0.9)
Hyperbilirubinemia	50 (45.5)	49 (44.5)	1 (0.9)
ALT increase	39 (35.5)	37 (33.6)	2 (1.8)
Hypertriglyceridemia	38 (34.5)	37 (33.6)	1 (0.9)
Lipase increase	28 (25.5)	20 (18.2)	8 (7.3)
ALP increase	23 (20.9)	23 (20.9)	0 (0)
Hypercholesterolemia	20 (18.2)	20 (18.2)	0 (0)
Hyperkalemia	18 (16.4)	16 (14.5)	2 (1.8)
Hyponatremia	15 (13.6)	14 (12.7)	1 (0.9)
Amylase increase	15 (13.6)	11 (10)	4 (3.6)
AST increase	13 (11.8)	12 (10.9)	1 (0.9)
Hypernatremia	8 (7.3)	8 (7.3)	0 (0)
Azotemia	8 (7.3)	8 (7.3)	0 (0)
Hypoalbuminemia	6 (5.5)	6 (5.5)	0 (0)

a Grade 3–5 adverse events occurring in <5% of patients were counted in the total frequency, but not listed in the table.

b Only adverse events occurring in ≥5% of patients at any grade were summarized.

ITT, intention to treat; ALT, alanine aminotransferase; ALP, alkaline phosphatase; AST, aspartate aminotransferase.

Anemia and thrombocytopenia occurred in 65 (59.1%) and 38 (34.5%) patients, respectively, and 7 (6.4%) and 10 (9.1%) of them were of grades 3–5. Grade 3–5 neutropenia was reported in 9 (8.2%) patients, including 2 (1.8%) cases of febrile neutropenia. Hypocalcemia and hyperbilirubinemia were the most common biochemical AEs, occurring in 62 (56.4%) and 50 (45.5%) patients, respectively. Hypertriglyceridemia and hypercholesterolemia were reported in 38 (34.5%) and 20 (18.2%) patients, respectively. Because routine monitoring of serum glucose and glycated hemoglobin levels was not mandated in the study protocol, the frequency of hyperglycemia and newly occurring, or worsening, diabetes mellitus could not be evaluated.

Transient dose interruptions or changes were made in 15 (13.6%) patients due to high‐grade AEs and 9 (8.2%) discontinued treatment before completion of the 2‐year study. Dropout causing AEs included thrombocytopenia (*n* = 3), neutropenia (*n* = 1), unstable angina (*n* = 1), hepatotoxicity (*n* = 1), acute viral hepatitis (*n* = 1), pancreatitis (*n* = 1), and infectious colitis (*n* = 1). Across the four MPNC groups, there were no significant linear trends in the frequency of total AEs, nonlaboratory AEs, hematological AEs, or biochemical AEs, either at any grade or at grades 3–5 (Table 5).

**Table 5:** Frequency of adverse events in the four MPNC groups

Adverse events	Low (*N* = 24)	Low‐intermediate (*N* = 23)	High‐intermediate (*N* = 24)	High (*N* = 23)	*P*‐value
Total, *n* (%)					
Any grade	20 (83.3)	21 (91.3)	24 (100)	17 (73.9)	0.556
Grade 3–5	9 (37.5)	9 (39.1)	8 (33.3)	5 (21.7)	0.226
Nonlaboratory abnormality, *n* (%)					
Any grade	17 (70.8)	17 (73.9)	22 (91.7)	16 (69.6)	0.7
Grade 3–5	3 (12.5)	0 (0)	3 (12.5)	4 (17.4)	0.353
Hematological abnormality, *n* (%)					
Any grade	13 (54.2)	15 (65.2)	17 (70.8)	13 (56.5)	0.759
Grade 3–5	6 (25)	4 (17.4)	4 (16.7)	1 (4.3)	0.064
Biochemical abnormality, *n* (%)					
Any grade	18 (75)	17 (73.9)	20 (83.3)	14 (60.9)	0.432
Grade 3–5	3 (12.5)	7 (30.4)	2 (8.3)	1 (4.3)	0.152

MPNC, mean plasma nilotinib concentration.

## Discussion

In ENESTKorea, we determined the efficacy and safety of nilotinib, administered at the currently approved dose (300 mg twice daily), in patients with Ph‐positive CML‐CP in South Korea. The results confirm findings from previous studies 5, 6, 7, showing excellent efficacy of nilotinib, with 56.2% of the enrolled patients achieving MR^4.5^ by 24 months (the primary endpoint), with tolerable safety profiles. Achievement of a deep molecular response (DMR), represented by MR^4.5^, is known as a key surrogate marker for a desirable long‐term prognosis in patients with CML. Patients who achieve DMR have better clinical outcomes, including decreased risks of progression and relapse, and longer survival than those who fail to achieve DMR 18, 19. Furthermore, select patients with durable (usually ≥2 years) DMR are potential candidates for attempting TKI discontinuation, given that they are under more frequent molecular monitoring than typically recommended for patients on active TKI therapy 20, 21.

We note that the cumulative rates of molecular responses were higher throughout the observed period in ENESTKorea than in the same‐dosage arms of previous phase 3 trials (ENESTnd and ENESTchina) and the phase 3b ENEST1st study 5, 6, 7. For example, the cumulative rate of MMR by 12 months was 80.6% (95% CI, 72.8–88.4%) in ENESTKorea versus 55%, 56%, and 68.9% in the ENESTnd, ENESTchina, and ESEST1st studies, respectively, and the cumulative rates of MR^4^ and MR^4.5^ by 24 months were 73.8% (95% CI, 64–83.7%) and 56.2% (95% CI, 44–68.3%), respectively, in ENESTKorea versus 39% and 25% in the ENESTnd study, and 55.2% and 38.6% in the ENEST1st study. These differences have probably arisen from the methodological measure implemented in ENESTKorea that censored patients on the last molecular assessment date if they were lost to follow‐up. In contrast, other studies considered patients with missing molecular assessments to be nonresponders, regardless of the reason. Since dropouts of patients due to reasons not related to the clinical outcomes of interest are common in clinical trials, treating these patients as nonresponders in a time‐to‐response analysis may underestimate the effectiveness of the investigated agent. Therefore, the cumulative response rates may better reflect the real‐world outcomes.

In ENESTKorea, analysis of the relationship between measured PNC levels and clinical outcomes has shown no significant exposure–outcome associations in terms of the cumulative rates of MMR, MR^4^, and MR^4.5^ or the frequency of AEs. These results imply that measuring the PNC level may provide no clinical benefit in CML patients. In contrast to the results from this study, previous studies showed that the administration of higher‐dose nilotinib was associated with more frequent AEs, such as cardiovascular events and fluid retention, and the steady‐state trough concentration of nilotinib was correlated with responses and laboratory abnormalities 7, 8, 9. Similarly, in CML patients treated with standard‐dose imatinib, the mean trough plasma drug level was significantly higher in those who achieved CCyR and/or MMR, than in those without CCyR and/or MMR, and the optimal sensitivity and specificity to discriminate patients with MMR were 77% and 71%, respectively 22. Negative exposure–outcome associations may be due to a lack of statistical power, owing to an insufficient sample size (*n* = 94). However, it should be noted that the guideline regarding time points of plasma sampling for PNC measurements (that recommended sampling before 10:00 am and before taking nilotinib on that day) was provided to the investigators but not specified in the study protocol, and data regarding the sampling time points were not collected. Therefore, we could not know whether the obtained PNC values were truly the trough levels. The substantial intra‐individual variation in PNC levels (Fig. S2) possibly reflects the various time points of plasma sampling. Uncontrolled timing of plasma sampling might have masked the true relationship between the PNC level and clinical outcomes. Therefore, future research with a larger sample size and tightly scheduled sampling is necessary to draw a firm conclusion on this topic.

Although the cross‐study comparison should be made with caution, patients were slightly older at enrollment in ENESTKorea (median age, 55 years) than in the nilotinib 300 mg twice daily arm of the ENESTnd trial and in the ENEST1st study (median age, 47 and 53 years, respectively). This contradicts the epidemiological data, suggesting CML patients are younger in Asians than in Western populations 23, 24, and implies that the patients enrolled in ENESTKorea better represent the elderly population, compared to previous studies. Although TKIs are well tolerated in elderly patients, old age is still among the predictors of poor outcome for CML, rendering elderly patients to be of major clinical concern 25. Therefore, results from ENESTKorea may provide a practical reference for real‐world practicing clinicians, who treat elderly CML patients.

This study has limitations. First, the study period was set to be too short (2 years) to show the long‐term efficacy and safety of nilotinib. Some patients did not complete the 2‐year study treatment and were lost to follow‐up due to various causes. These limitations have hindered precise estimation of the clinical outcomes and reduced statistical power. Second, as mentioned above, the sampling time for plasma used for the PNC measurements was not strictly controlled. However, the impact of the high variability in PNC levels per patient on the results was minimized through averaging all measured PNC levels in each patient to obtain the MPNC values that were used in analysis. Third, no outcome data (including qRT–PCR assessment results and safety data) were collected after discontinuation of the study treatment, meaning that analysis was limited to patients on treatment. Fourth, the spleen size was not recorded at baseline. Because this information is necessary to calculate the Sokal 26, Hasford 27, or European Treatment and Outcome Study risk scores 28, the three most widely used risk scoring systems for CML, we could not analyze the relationship between the baseline risk grades of patients and treatment outcomes.

Despite the limitations, to the best of our knowledge, ENESTKorea is the first phase 4 study, evaluating the efficacy and safety of nilotinib and the clinical utility of directly measured PNC levels in CML‐CP patients. Overall, nilotinib is a highly effective therapeutic option for CML‐CP patients with manageable AEs and no newly emerging safety concerns. The measurement of PNC levels, however, seems to provide no information regarding efficacy or safety outcomes and is thus not considered clinically useful.

## Ethics and Registration

The study protocol and consent forms were approved by the institutional review board at each participating institution. Written informed consent was provided by all participants. All procedures were carried out in accordance with the ethical standards of each institutional research ethics committee and the Helsinki Declaration (revised in 2013; World Medical Association). This study is registered with clinicaltrials.gov, Number NCT03332511.

## Conflict of Interest

The authors declare no conflict of interests.

## Supporting information


**Data S1**. Exclusion criteria.**Data S2**. Guideline for resuming nilotinib treatment after transient interruption.**Figure S1**. Histogram of the plasma concentration of nilotinib (*N* = 296).**Figure S2**. Per‐patient distributionof the plasma nilotinib concentration (PNC).**Figure S3**. Trendin the plasma nilotinib concentration levels according to time after enrollment.
