# Supplementary material for: A phase 4 study of nilotinib in Korean patients with Philadelphia chromosome‐positive chronic myeloid leukemia in chronic phase: ENESTKorea
Source: Cancer Med. 2018 Mar 25;7(5):1814–23. doi: 10.1002/cam4.1450 (PMC5943463; doi:10.1002/cam4.1450)
Supplement: Supplementary file 1 — Data S1. Exclusion criteria.Data S2. Guideline for resuming nilotinib treatment after transient interruption.Figure S1. Histogram of the plasma concentration of nilotinib (N = 296).Figure S2. Per‐patient distributionof the plasma nilotinib concentration (PNC).Figure S3. Trendin the plasma nilotinib concentration levels according to time after enrollment. [file CAM4-7-1814-s001.pdf]

Supplementary Methods S1

- Exclusion criteria

1. CML with atypical *BCR-ABL1* transcripts (transcripts other than e13a2 or e14a2)
2. Previous treatment with myelosuppressive agents except hydroxyurea and anagrelide
3. Previous treatment with TKI for over two weeks
4. Previous hematopoietic stem cell transplantation
5. Previous irradiation involving 25% or more of the bone marrow tissue
6. Cytopathologically confirmed central nervous system involvement of CML (cerebrospinal fluid analysis was not mandatory if not clinically indicated)
7. Eastern Cooperative Oncology Group performance status ≥ 3
8. Cardiac abnormality including a corrected QT interval ≥ 480 milliseconds, complete left bundle branch block, permanent pacemaker implantation, congenital long QT syndrome, history of tachyarrhythmia requiring treatment, clinically significant resting bradycardia, history of acute coronary syndrome within 12 months, and decompensated congestive heart failure
9. Organ dysfunction defined by total serum bilirubin levels ≥ 1.5 × the upper limit of the normal range (ULN), creatinine ≥ 1.5 × ULN, aspartate or alanine aminotransferase ≥ 2.5 × ULN, amylase or lipase ≥ 1.5 × ULN and alkaline phosphatase ≥ 2.5 × ULN not directly related to the CML
10. Active and uncontrolled malignancy other than CML
11. Uncontrolled hypertension and/or diabetes
12. Active and uncontrolled infection
13. Major surgery within two weeks or incomplete recovery from the previous surgery
14. Congenital or acquired bleeding tendency
15. Impaired gastrointestinal absorption
16. History of small bowel resection or bypass surgery
17. History of acute pancreatitis within 12 months or chronic pancreatitis

18. Concomitant administration of strong irreplaceable CYP3A4 inhibitors or inducers, QT-prolonging agents, or coumarin derivatives
19. Any other uncontrolled medical conditions that would present substantial safety risks or compromise compliance with the study treatment

Supplementary Methods S2

- Guideline for resuming nilotinib treatment after transient interruption

Following the first and second occurrence of grade 3 or 4 hematological adverse events (AEs), nilotinib was resumed without dose modification upon improvement to grade ≤ 2, while one-week and one-month rests were recommended before resuming treatment at the third and fourth occurrence, respectively. Treatment was discontinued indefinitely after the fifth occurrence. Following the first occurrence of grade 2 non-hematological AEs, nilotinib was resumed at the same dose upon improvement to grade 1. After a recurrence of grade 2, or the first occurrence of grade 3 or 4, the dose was reduced to 300 mg once daily, upon improvement to grade 1. Treatment was discontinued indefinitely after a recurrence of grade 3 or 4 non-hematological AEs.

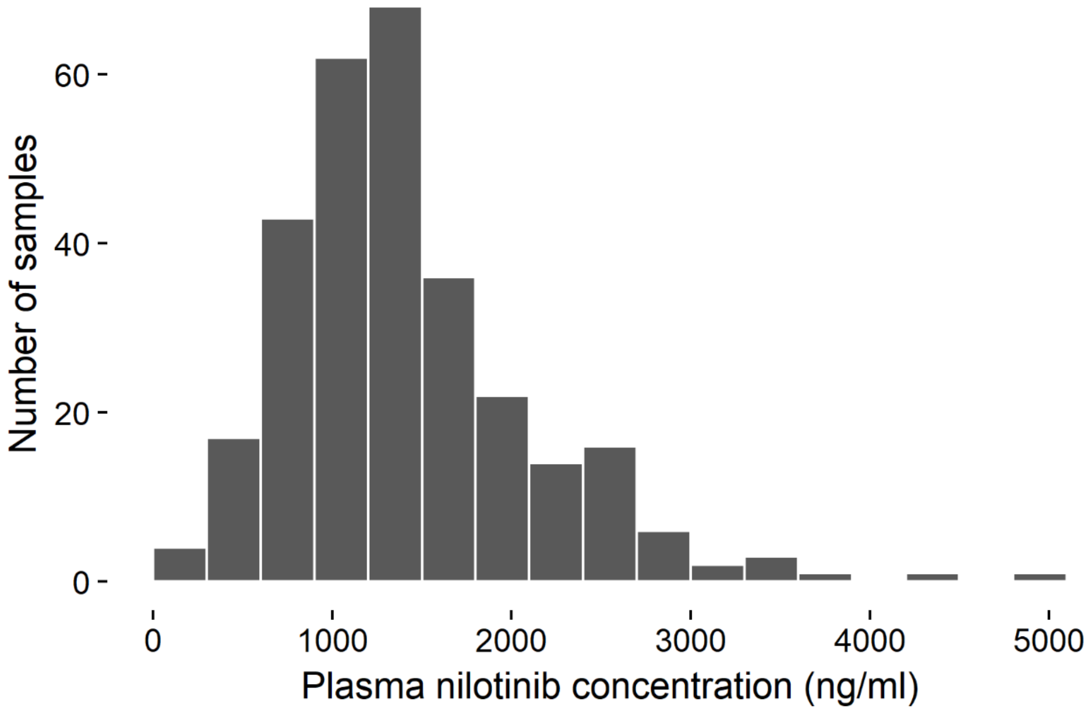

Supplementary Figure 1. Histogram of the plasma concentration of nilotinib (N = 296).

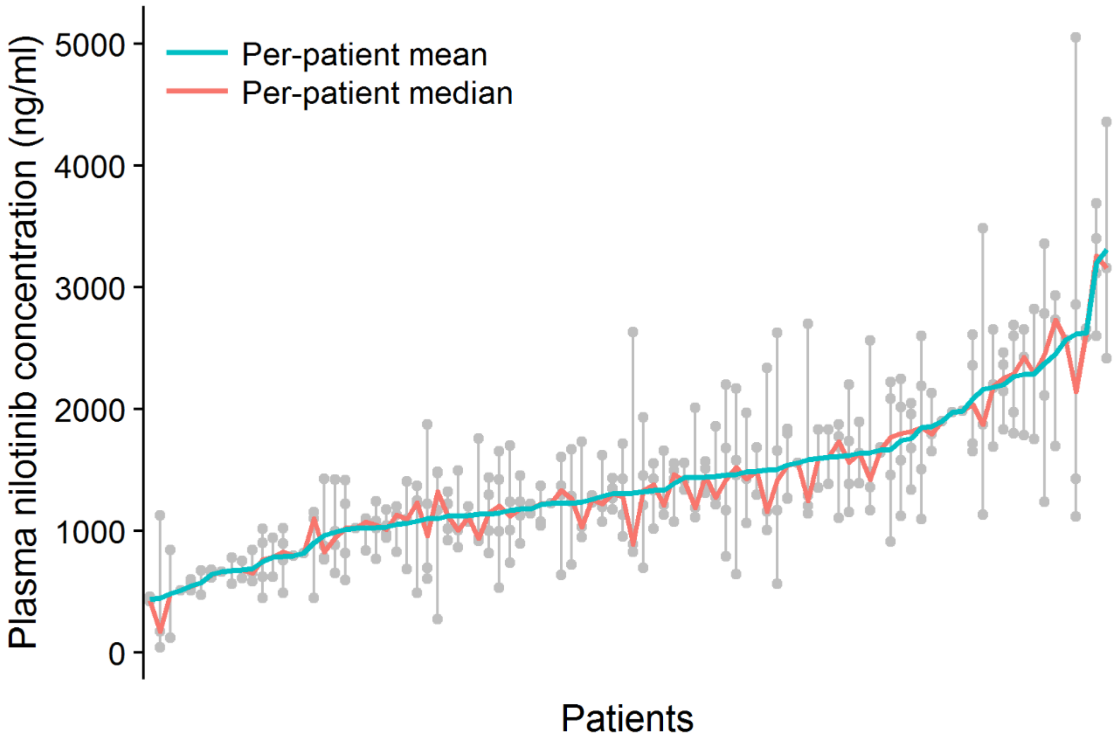

Supplementary Figure 2. Per-patient distribution of the plasma nilotinib concentration (PNC). Patients are arranged in ascending order of the arithmetic mean PNC (MPNC) from left to right. Each sample is represented by a gray dot and samples from the same patient are connected by a gray line.

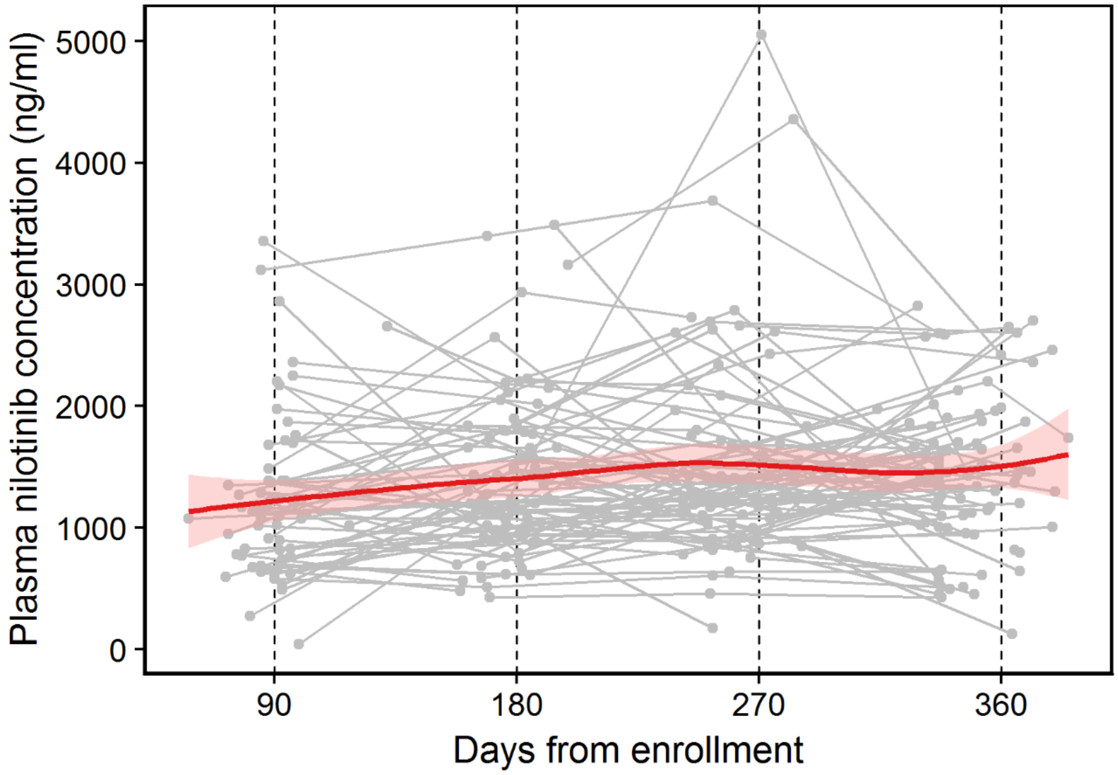

Supplementary Figure 3. Trend in the plasma nilotinib concentration levels according to time after enrollment. Each sample is represented by a gray dot and samples from the same patient are connected by a gray line. The red line and pink shading indicate a locally weighted scatterplot smoothing curve and the corresponding 95% confidence interval, respectively.
